# The Evolution and Prognostic Role of Tumour-Infiltrating Lymphocytes and Peripheral Blood-Based Biomarkers in Inflammatory Breast Cancer Patients Treated with Neoadjuvant Chemotherapy

**DOI:** 10.3390/cancers13184656

**Published:** 2021-09-16

**Authors:** Christophe Van Berckelaer, Iris Vermeiren, Leonie Vercauteren, Charlotte Rypens, Gizem Oner, Xuan Bich Trinh, Wiebren A. A. Tjalma, Glenn Broeckx, Emmanuelle Charafe-Jauffret, Steven Van Laere, François Bertucci, Cecile Colpaert, Peter A. van Dam

**Affiliations:** 1 Translational Cancer Research Unit, GZA Hospitals, 2000 Antwerp, Belgium; rypens.charlotte@hotmail.com; 2 Multidisciplinary Breast Clinic, Unit Gynaecologic Oncology, Antwerp University Hospital (UZA), 2650 Edegem, Belgium; iris.vermeiren@student.uantwerpen.be (I.V.); Leonie.Vercauteren@student.uantwerpen.be (L.V.); onergizem@hotmail.com (G.O.); XuanBich.Trinh@uza.be (X.B.T.); wiebren.tjalma@uza.be (W.A.A.T.); peter.vandam@uza.be (P.A.v.D.); 3 Center of Oncological Research (CORE), MIPRO, IPPON, University of Antwerp, 2610 Wilrijk, Belgium; Glenn.broeckx@uza.be (G.B.); steven.vanlaere@uantwerpen.be (S.V.L.); cecile.colpaert@telenet.be (C.C.); 4 Department of General Surgery, Kocaeli University, Kocaeli 410040, Turkey; 5 Department of Pathology, UZA, Antwerp University Hospital, 2650 Edegem, Belgium; 6 Predictive Oncology Team, Centre de Recherche en Cancérologie de Marseille (CRCM), INSERM, CNRS, Institut Paoli-Calmettes, Aix-Marseille Université, 13273 Marseille, France; JAUFFRETE@ipc.unicancer.fr (E.C.-J.); bertuccif@ipc.unicancer.fr (F.B.)

**Keywords:** inflammatory breast cancer (IBC), neo-adjuvant chemotherapy (NACT), stromal tumour-infiltrating lymphocytes (sTIL), immune response, lymphocyte-to-monocyte ratio (LMR), neutrophil-to-lymphocyte ratio (NLR), platelet-to-lymphocyte ratio (PLR)

## Abstract

**Simple Summary:**

Inflammatory breast cancer (IBC) is a rare and aggressive form of breast cancer (BC) in which higher levels of stromal tumour-infiltrating lymphocytes (sTIL) before neo-adjuvant chemotherapy (NACT) are associated with a better outcome. The role of sTIL in patients with residual disease (RD) after NACT is not clearly established. In this study, we showed that a high number of sTIL after NACT was associated with a worse outcome. Furthermore, we also demonstrated that sTIL decreased more during NACT in IBC compared to subtype-matched non-IBC patients (nIBC) treated with NACT. We also looked at the effect of NACT on some peripheral immune markers. Unlike the sTIL, we could not demonstrate a prognostic effect of these markers after NACT and their change was not significantly different between IBC and nIBC, indicating that the effect of NACT on the peripheral immune response seems to be similar in IBC and nIBC.

**Abstract:**

Introduction: Inflammatory breast cancer (IBC) is a rare but aggressive form of breast cancer (BC) in which the (prognostic) role of stromal tumour-infiltrating lymphocytes (sTIL) and the peripheral circulating immune cells in patients with residual disease (RD) after neo-adjuvant chemotherapy (NACT) is not clearly established. Methodology: To describe the evolution of sTIL and some peripheral inflammation markers (Neutrophil-to-lymphocyte ratio, Platelet-to-lymphocyte ratio and Lymphocyte-to-monocyte ratio) after NACT in IBC, we retrospectively collected clinicopathological variables for 125 stage III IBC patients. sTILs were scored by three different researchers on an H&E slide of the mastectomy specimen. A cohort of subtype-matched non-IBC breast cancer patients (nIBC) treated with NACT was included for comparison. Results: There was no significant difference in the pre- and posttreatment sTIL scores between IBC and nIBC and in both groups the number of sTIL was significantly lower after NACT. However, the IBC phenotype did correlate with a stronger decrease of sTIL after NACT (OR: 0.25, 95% CI: 0.073–0.76, *p* = 0.018). The change in the peripheral immune markers was not significantly different between IBC and nIBC. After NACT, 75 patients had residual disease. In this group, a high number of sTIL before NACT (HR: 0.23, 95% CI: 0.05–1.02, *p* = 0.05) was prognostic for a longer OS, while a low number of sTIL after NACT (HR: 0.33, 95% CI: 0.11–0.98, *p* = 0.046) and a low residual cancer cellularity (HR: 0.20, 95% CI: 0.08–0.52, *p* < 0.001) was associated with a longer DFS. Conclusions: IBC is associated with a significantly stronger decrease of sTIL after NACT compared to nIBC. Furthermore, a high number of sTIL after NACT was associated with a worse prognosis in IBC.

## 1. Introduction

Inflammatory breast cancer (IBC) is a rare form of breast cancer (BC), characterised by a rapid local progression and fast dissemination of the tumour. This is reflected in the poor prognosis of IBC with a five-year overall survival rate of 61% for stage III and 21% for stage IV disease [1,2]. Growing evidence indicates that infiltrating immune cells in the tumour micro-environment (TME) of IBC not only plays an important role in an anti-tumour response, but also that some of the unique biological features of IBC might be attributed to a specific but suppressed immune micro-environment [3,4,5]. 

Stromal tumour-infiltrating lymphocytes (sTIL) give a valuable insight into the anti-tumour response and have been correlated with prognosis in the more aggressive and proliferative triple negative (TN) and human epidermal growth factor receptor 2 (HER2+) subtypes of breast cancer [6]. Furthermore, higher levels of sTIL are associated with increased pCR rates after neo-adjuvant chemotherapy (NACT) in both non-IBC (nIBC) and IBC [7]. However, the prognostic role of sTIL in patients without pCR after NACT remains unclear.

In two studies evaluating TNBC patients, more sTIL in residual disease (RD) after NACT was associated with an improved recurrence-free survival (RFS) and overall survival (OS) [8,9]. In contrast, Hamy et al. showed no association with outcome in TNBC and an even worse prognostic impact of high post-NACT TIL levels in the cohort of HER2-positive BC patients [10]. Only one study looked at the evolution of sTIL in TN IBC and reported an association between a shorter RFS and an NACT-induced increase in sTIL [11]. Furthermore, while most studies report a decrease of mean sTIL after NACT, some report an increase [12]. In Table 1, an overview of the most recent articles about the evolution of sTIL after NACT is summarised. 

The role of the peripheral circulating immune cells on (inflammatory) breast cancer is less established. In a large meta-analysis, including 8563 breast cancer patients, a significant negative prognostic effect of an elevated peripheral neutrophil-to-lymphocyte (NLR) ratio on OS and RFS was observed [13]. However, the correlation between a higher NLR and worse outcome in the neo-adjuvant setting seems to be less clear [14]. Besides the NLR, an elevated platelet-to-lymphocyte ratio (PLR) [15,16] and a lower lymphocyte-to-monocyte ratio (LMR) [17] are other peripheral inflammation markers associated with a worse outcome in breast cancer. Both a low PLR [18] and a low NLR [19] were also found to be predictive factors for pCR after NACT, although other studies failed to confirm this [14]. We previously demonstrated that an elevated NLR or PLR in IBC is also associated with a worse outcome and that the elicited peripheral immune response in IBC seems similar to the response in nIBC [20]. However, even though the number of peripheral immune cells is comparable, the functional state of these leukocytes might be impaired and their response to NACT different [21]. 

**Table 1:** Evolution of sTIL in breast cancer after NACT and the prognostic impact.

Authors (Year of Publication)	N	Subtype of BC	Type of NACT	Number of sTIL (Pre & Post-NACT)	Change in sTIL after NACT	Correlation between Post-NACT sTIL and Clinicopathological Parameters	Prognostic Effect of Post-NACT sTIL
Campedel et al. (2020) [11]	31	TN IBC	Anthracycline Taxane-based	Median Pre: 10% Median Post: 1.5%	Median delta TIL was −9% (−50% up to +40%)	Not reported	A positive delta TIL was significantly associated with a decrease of EFS. HR: 1.88; 95% CI: 1.05–3.35; *p* = 0.01
Lee et al. (2020) [22]	104	TNBC	Anthracycline Taxane-based	Mean Pre: 23.3 ± 20.8% Mean Post: 17.1 ± 24.2%	Mean delta TIL: −2.69% - Decrease: 41.3% - No change: 30.8% - Increase: 27.9%	pT stage after NACT	RFS: Positive, HR:0.979, 95% CI: 0.961–0.997, *p* = 0.023 Change in sTIL level is associated with an increase in RFS: Decrease vs. no change: HR: 0.439, 95% CI: 0.228–0.846, *p* = 0.014; Increase vs. no change HR: 0.359, 95% CI: 0.158–0.814 *p* = 0.014)
Ochi et al. (2019) [23]	209	80 TNBC 129 HER2+	Anthracycline Taxane-based	Mean TNBC-Pre: <10%: 31.3% ≥10%: 68.7% TNBC-Post: <10%: 41.8% ≥10%: 58.2% Mean HER2+-Pre: <10%: 38.8% ≥10%: 61.2% HER2+-Post: <10%: 66.7% ≥10%: 33.3%	TNBC: - Increase: 12.7% - No change: 70.9% - Decrease: 16.4% HER2+ BC: - Increase: 4.0% - No change: 76.0% - Decrease: 20.0%	Not reported	RFS: Positive, HR: 2.836, 95% CI: 0.951–8.457, *p* = 0.06 (only in TNBC) The change in TILs was not associated with RFS.
Luen et al. (2019) [8]	375	TNBC	Anthracycline Taxane-based (in 62%)	Median Post: 20%	Increase: 48% Decrease: 47% Mean delta TIL: −3%	sTIL levels were significantly lower with increasing post-NACT tumour size and nodal stage, but did not differ by RCB class.	RFS: Positive, HR: 0.86; 95% CI: 0.79–0.92; *p* < 0.001 OS: Positive, HR: 0.87; 95% CI: 0.80–0.94; *p* < 0.001
Hwang et al. (2019) [24]	204	All subtypes (25% HER2, 31% TN)	Taxane Platinum-based	Median Pre: 14.6% Median post: 10.2%.	Decrease: 44% No change: 39% Increase: 17%	A positive delta TIL was associated with smaller residual tumour size, negative nodal status, and lower RCBs.	High post-NACT sTIL levels (>50%) were associated with longer BCSS and DFS: BCSS: HR: 6.57, 95% CI: 0.87–19.57 *p* = 0.005 DFS: HR: 2.24, 95% CI: 0.81–5.48 *p* = 0.025
Hamy et al. (2019) [10]	718	All subtypes (320 TN, 175 HER2+, 223 HR+ BC)	85.0% Anthracycline Taxane-based 8.6% Anthracycline-based 3.2% Taxane-based	Mean pre-NACT: 24.2% (luminal: 16.2%; TNBC: 28.5%; HER2+: 26.5%; *p* < 0.001) Mean post-NACT: 13% (TNBC: 15.4%; luminal: 11.3%; HER2+: 10.9%, *p* < 0.001)	Decrease: 61.6% No change: 17.7% Increase: 20.7%	High post-NACT sTIL levels were associated with cellularity in HER2+ BC (*p* < 0.001)	High post-NACT sTIL levels were associated with impaired DFS in HER2-positive breast cancers (HR 1.04, CI 1.02–1.06, *p* = 0.001), but not in luminal tumours or TNBC.
Zhang et al. (2018) [25]	58	TNBC	Anthracycline Taxane-based	Two categories: (cut-off 60%) PRE - High sTIL: 7 - Low sTIL: 22 POST - High sTIL: 5 - Low sTIL: 24	No statistically significant difference before and after NACT.	Not reported	Not reported
Pelekanou et al. (2017) [12]	58	All subtypes (79.3% HR+)	56% Anthracycline Taxane-based	Median Pre: 5% Median Post: 7,5%	Delta sTIL: 5%. Trend towards increase in sTIL (*p* = 0.09)	Post-NACT sTIL were higher in ER-negative tumours (12.5%) than in ER-positive tumours (5%).	A positive delta TIL was significantly associated with an increase of EFS. HR: 3.9; CI 1.17–15.39; *p* = 0.02
Castaneda et al. (2016) [26]	80	TNBC	88% Anthracycline Taxane-based 9% Anthracycline -based 3% Taxane-based	Median Pre: 40% Median Post: 20%	Statistically significant decrease after NACT in median TIL percentage (*p* < 0.0002)	None	None
Dieci et al. (2014) [9]	278	TNBC	48%Anthracycline-based 45% Anthracy cline/taxane-based	Two categories: (cut-off 60%) - High sTIL: 27 - Low sTIL: 251	Pre-NACT slides available for 19 of the 27 patients with High sTIL RD. Change in sTIL: - Decrease: 1 patient - Increase: 18 patients	The presence of high TIL in RD was significantly associated with absence of metastatic axillary nodes and small tumour size (≤2 cm).	RFS: Positive, HR: 0.86, CI 0.79–0.92, *p* < 0.001 OS: Positive, HR: 0.86, CI 0.77–0.97, *p* = 0.01

In this study, we describe the evolution of sTIL and some peripheral inflammation markers after NACT in IBC. We compare this evolution with a control cohort of nIBC patients to discover IBC-specific features and examine the prognostic value of sTIL and the peripheral blood-based biomarkers after the completion of NACT.

## 2. Materials and Methods

### 2.1. Study Population 

We previously reported a retrospective cohort of IBC patients who had their initial diagnosis and complete treatment at GZA Hospital Sint-Augustinus, Antwerp University Hospital or Institut Paoli-Calmettes, Marseille, France between 1 June 1996, and 31 December 2016 [4,20]. In this study we analysed all stage III patients (n = 125) that were diagnosed based on the clinical IBC definition [27] with pathological confirmation of invasive carcinoma and complete hospital records. Patients who were not treated with an anthracycline/taxane-based NACT regime or who did not undergo a radical mastectomy after completion of NACT were not included in this study. During the study interval, systemic therapy changed, but most HER2+ patients received targeted therapy (n = 30/44). Pathological complete response (pCR) was defined as the absence of residual invasive carcinoma in the resected breast specimen and in all sampled regional lymph nodes after completion of NACT. Out of the 75 patients that had residual disease (RD: no-pCR), only 52 were included to compare pre- and post-operative tumour slides because seven had only residual lymphatic disease and 16 had no more post-operative slide-containing tumours in the pathology archive. Estrogen (ER) and progesterone receptor (PgR) expression were assessed using validated immunohistochemical tests and were defined as positive if Allred score ≥ 3/8. Tumour samples were considered HER2-positive when a fluorescence in situ hybridisation (FISH) test documented amplification.

A retrospective cohort of 134 advanced and subtype-matched non-IBC breast cancer patients (nIBC) treated with NACT was included in this study to compare the evolution of sTIL, NLR, PLR and LMR between IBC and nIBC. Other inclusion criteria included an anthracycline/taxane-based NACT regimen followed by a tumorectomy or mastectomy. Most HER2+ patients received Trastuzumab (n = 43/48). This cohort was sampled at random, using the cancer registry from the Antwerp University Hospital in the timeframe between 1 January 2006 and 31 December 2017 to match the same period in which most IBC cases were diagnosed. This study was approved by the ethical committee of the Antwerp University Hospital (Filenumber: 16/33/338).

### 2.2. Blood-Based Biomarkers

The peripheral blood cell count was determined both at the moment of diagnosis [20] and right before surgery after completion of the NACT regimen. This pre-operative blood test was performed as part of the routine management of the patient. The NLR was computed by dividing the absolute neutrophil count by the absolute lymphocyte count. The absolute number of platelets was divided by the absolute number of lymphocytes to calculate the PLR and the LMR was defined as the absolute lymphocyte count divided by the absolute monocyte count.

### 2.3. Stromal Tumour-Infiltrating Lymphocytes (sTIL) and Cellularity in the Residual Tumour Bed

Scoring of the sTIL after NACT was done on haematoxylin and eosin (H&E) stained 5-μm sections of formalin-fixed paraffin-embedded (FFPE) tumour tissue by three different researchers (CC, LV, IV). The specific recommendations by the International TILs Working Group for scoring sTIL in residual tumour tissue after NACT were applied [28]. In short, a semi-quantitative assessment of the percentage of stromal compartment invaded by sTIL in all areas containing invasive tumour cells on the H&E slide containing the most residual invasive tumour was made. Furthermore, for all IBC patients with pCR, sTIL were also evaluated in the tumour bed. 

The interclass correlation coefficient (ICC) (two-way, agreement model) for the sTIL, scoring between the different researchers was 0.728 (95% CI: 0.675–0.774, *p* < 0.001), showing a good agreement. A mean score was calculated and used both as continuous and categorical variable: <10% (category 1), ≥10–40% (category 2), and ≥40% (category 3). In case of discrepant results a consensus score was determined after consulting an extra pathologist (GB).

Pathology reports were reviewed, but because of missing data it was impossible to calculate the residual cancer burden (RCB) for the IBC cohort. Therefore, we can only report the cellularity: the proportion of the residual tumour bed occupied by invasive cancer cells (%CA) after microscopic evaluation of the slide with the most residual tumour on which the sTIL were also scored. 

### 2.4. Statistical Analysis

Statistical analysis was performed using R studio (Version 1.1.463 using the following packages: dplyr, tidyr, irr, survival, survminer and ggplot2) [29] and cases with missing data were maintained in the database but excluded from the statistical analyses on a per test basis. For comparison between the two IBC and nIBC cohorts, a Pearson Chi2 test was used for the categorical parameters and a Mann–Whitney U test for continuous parameters. Evolution of the parameters before and after NACT was assessed with a Paired Wilcoxon signed-rank test. Significant parameters in univariate analysis were included in a multivariate logistic regression model. For dichotomisation, the median value was used. Two survival endpoints were measured: recurrence-free survival (RFS) defined as the interval from the date of pathological diagnosis to the date of cancer recurrence and overall survival (OS) defined as the interval between pathological diagnosis and death. Patients that were not relapsed or dead at the time of analysis were censored at the date of their last follow-up visit with a last update of the survival data on 31 December 2019. Survival curves were estimated with Kaplan–Meier curves and compared using the log-rank test. To evaluate the effects of all significant clinicopathological variable factors on survival, a multivariate cox proportional hazard model was used. *p*-values were calculated two-sided and considered statistically significant when less than 0.05.

## 3. Results

### 3.1. Patient Characteristics

Patient and tumour characteristics are described in Table 2. As expected, most of the IBC patients presented with a hormone receptor (HR) positive carcinoma (n = 75/125, 60.0%) and 50 patients had pCR after NACT (40.0%). Besides having histologically more poorly differentiated tumours (*p* = 0.001) and a higher stage (*p* < 0.001), inherent to the definition of IBC, no significant clinicopathological differences between the IBC and molecular subtype-matched nIBC cohorts were observed. 

A summary of the continuous parameters can be found in Table 3. There was no significant difference in the pre-treatment median sTIL score between IBC (12.5%, range: 1–80%) and nIBC (10%, range: 1–85%), nor in the median sTIL score after NACT (IBC: 4%, range 1–90% versus nIBC: 5%, range 1–60%). The NLR was comparable between IBC and nIBC, both before and after the NACT. However, the PLR after NACT was higher in the nIBC cohort (IBC: 204, range 51.5–840 versus nIBC: 274, range 43–1006, *p* = 0.03). Interestingly, the LMR was significantly higher in the nIBC stage compared to IBC before NACT (IBC: 3.43, range 1.0–9.5 versus nIBC: 4.35, range 0.69–23.7, *p* < 0.001) and significantly lower after (IBC: 2.28, range 0.79–7.2 versus nIBC: 1.74, range 0.52–13.9, *p* = 0.03). However, in a multivariate model, these differences between IBC and nIBC were not significant. 

### 3.2. Evolution of sTIL after NACT

**Figure 1:**
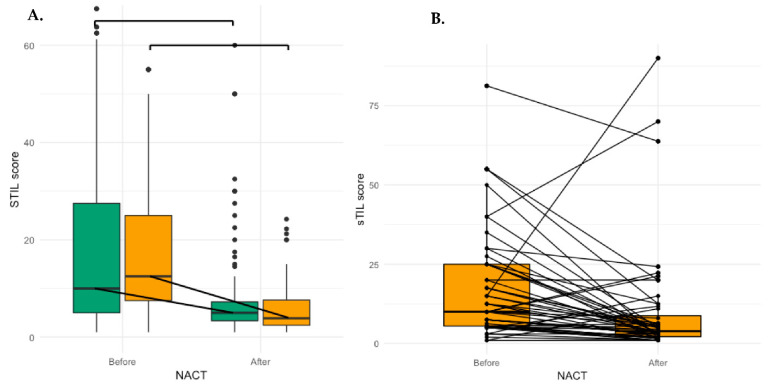

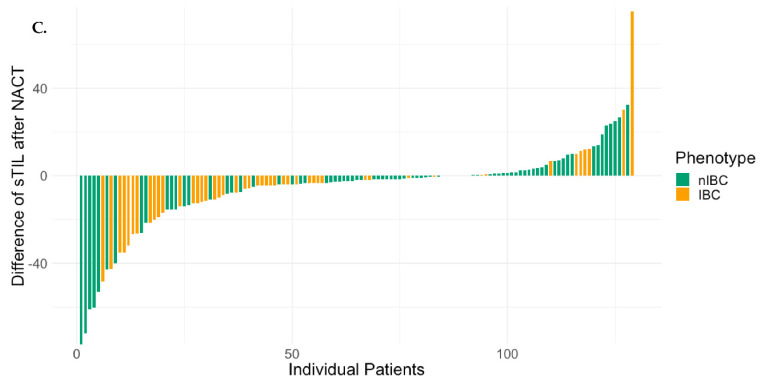
(**A**) Boxplot graph of the evolution of sTIL after NACT: In both IBC (median δsTIL: −4.5%, *p* < 0.001) and nIBC (median δsTIL: −1.25%, *p* = 0.06) the number of sTIL are lower after NACT (**B**) Boxplot depicting the evolution of median sTIL after NACT in individual IBC patients: Out of 50 patients, 7 had an increase, 21 had no change and 22 patients had a decrease. (**C**) Waterfall plot of sTIL difference (δsTIL) in all patients. IBC had more often a decrease than nIBC patients (*p* = 0.044). nIBC: non-inflammatory breast cancer, IBC: inflammatory breast cancer, sTIL: stromal tumour infiltrating lymphocytes, NACT: neo-adjuvant chemotherapy.

All IBC patients with pCR had less than 1% sTIL in the tumour bed area and were not included in further analyses. In both the IBC (median δsTIL: −4.5%, *p* < 0.001) and nIBC (median δsTIL: −1.25%, *p* = 0.06) cohorts, the number of sTIL was predominantly lower after NACT (Figure 1 and Figure S1) but this decrease was significantly higher in the IBC cohort (*p* = 0.005) (Table 3). We also looked at the data in HR+ (n = 33, *p* = 0.03), HER2+(n = 13, *p* < 0.001) and TNBC (n = 11, *p* < 0.001) patients and found a significant decrease for all subtypes before and after NACT. When compared to the nIBC cohort, this decrease was significantly higher in TN IBC (*p* = 0.007), borderline significant (*p* = 0.06) in the HR+ IBC and not significant in HER2+ IBC (*p* = 0.37). In a multivariate model including all patients, the IBC phenotype correlated with a stronger decrease of sTILs (<−2.5%) after NACT (OR: 0.25, 95% CI: 0.073–0.76, *p* = 0.018, Table 4). In a model with only IBC patients, a stronger decrease of sTIL correlated with a high number of sTIL before NACT (OR: 0.027, 95% CI: 0.001–0.19, *p* = 0.002) and a low number of sTIL after NACT (OR: 24.02, 95% CI: 3.60–493.67, *p* = 0.006). 

### 3.3. Evolution of Peripheral Blood-Based Biomarkers after NACT

**Figure 2:**
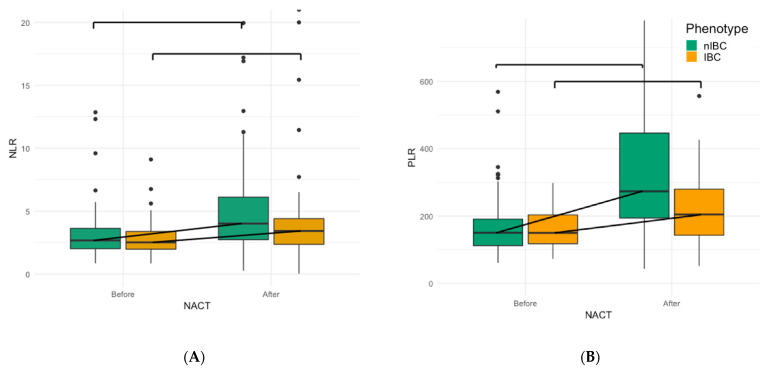
(**A**) Boxplot graph of the increase of the NLR after NACT in both IBC (*p* = 0.012) and nIBC (*p* < 0.001) (**B**) Boxplot graph of the increase of the PLR after NACT in IBC (*p* < 0.001) and nIBC (*p* < 0.001). Comparison between nIBC (green) and IBC (orange) was done using Wilcoxon signed-rank test. nIBC: non-inflammatory breast cancer, IBC: inflammatory breast cancer, NACT: neo-adjuvant chemotherapy, NLR: neutrophil-lymphocyte ratio, PLR: platelet-lymphocyte ratio.

The NLR significantly increased after NACT in both IBC (*p* = 0.012) and nIBC (*p* < 0.001); however, there was no significant difference in increase between IBC and nIBC (IBC: 0.52, range: −3.51–15.9 vs. nIBC: 1.05, range: −11–56.8, *p* = 0.2792) (Figure 2A). The PLR also increased both in IBC (*p* < 0.001) and nIBC (*p* < 0.001), but the increase was significantly higher in nIBC (IBC: 55.8, range: −127–343 vs. nIBC: 117, range: −308–847, *p* = 0.003). The LMR decreased both in IBC (*p* < 0.001) and nIBC (*p* < 0.001). This decrease was significantly higher in nIBC (IBC: −1.24, range: −1.06–−4.54 vs. nIBC: −2.81, range: −2.83–−20.6, *p* < 0.001). However, in a multivariate model there was no association between the IBC phenotype and a stronger decrease of the LMR or a stronger increase of the PLR. 

### 3.4. Parameters Associated with Lower sTIL after NACT

In the overall cohort (including both IBC and nIBC patients) the number of sTIL after NACT seemed to largely depend on the number of sTIL before NACT (OR: 2.66, 95% CI: 1.19–6.20, *p* = 0.019) and the residual cancer cellularity (OR: 3.50, 95% CI: 1.60–7.89, *p* = 0.004) (Tables S1 and S2). In the IBC cohort, the number of sTIL after NACT was only significantly associated with higher residual cancer cellularity (OR: 11.64, 95% CI: 2.99–55.29, *p* < 0.001) (Table 5). 

### 3.5. Prognostic Effects of sTIL and Peripheral Blood-Based Biomarkers

Out of 125 IBC patients, 40% (n = 50) reached pCR after NACT and a higher pre-NACT sTIL score (OR: 2.32 95% CI: 0.97–5.77, *p* = 0.06) tended to correlated with a better rate of pCR independently of molecular subtype (Figure S2 and Table S3). No peripheral blood-based biomarker was associated with a better response to NACT. 

**Figure 3:**
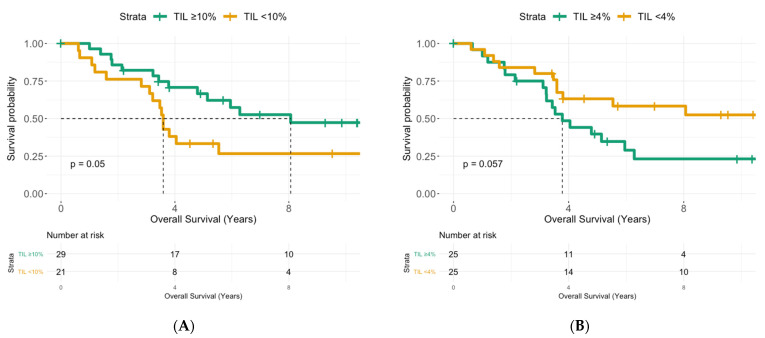

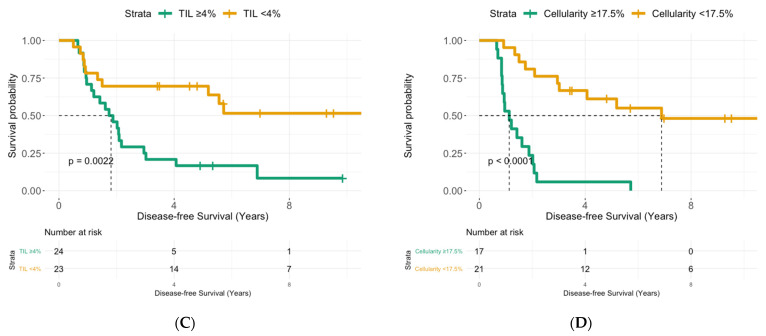
Kaplan–Meier curves for OS (**A**,**B**) and DFS (**C**,**D**) in IBC. A. Patients with ≥10% sTIL before NACT have a significant longer OS: Median survival of 8.07 year (95% CI: 5.14–NR) vs. 3.59 year (95% CI: 3.11–NR), *p* = 0.05 B. More sTIL after NACT (≥4%) is associated with a shorter OS: Median survival of 3.78 year (95% CI: 3.22–NR) vs. NR year (95% CI: 3.80–NR), *p* = 0.057. C. Patients with ≥4% sTIL after NACT have a significant shorter DFS: Median survival of 1.81 year (95% CI: 1.21–2.95) vs. NR year (95% CI: 5.19–NR), *p* = 0.002. D. A higher residual cellularity (≥17.5%) in the tumour bed is associated with a shorter DFS: Median survival of 1.13 year (95% CI: 0.88–2.02) vs. 6.88 year (95% CI: 3.02–NR), *p* < 0.001.

In the group of IBC patients without pCR after NACT, the median follow-up was 10.4 years (95% CI: 5.71–13.9). Median OS was 5.14 years (95% CI: 3.59–Not reached, NR) and median DFS was 2.17 years (95% CI: 1.61–6.88). In six patients (13.0%), local disease was the first sign of recurrence, while 24 patients (51.1%) presented with distant metastases. A longer OS was associated with a higher number of sTIL before NACT, lower residual cancer cellularity, minimal nodal disease and borderline significantly with a lower post-NACT sTIL score. In a multivariate model, only the number of sTIL before (HR: 4.47, 95% CI: 1.37–14.5, *p* = 0.01) and after NACT (HR: 0.23, 95% CI: 0.05–1.02, *p* = 0.05) remained significant (Table 6, Figure 3A,B).

In univariate analysis, a decrease in sTIL was associated with longer DFS (*P=* 0.036, Table 7, Figure S3), but in the multivariate model only a high number of sTIL after NACT (HR: 0.33, 95% CI: 0.11–0.98, *p* = 0.046) and a higher residual cancer cellularity (HR: 0.20, 95% CI: 0.08–0.52, *p* < 0.001) remained associated with a shorter DFS (Table 7, Figure 3C,D). 

Finally, we repeated this analysis for the different molecular subtypes, albeit the HR and HER2 status not being significant for OS or DFS in the IBC cohort. In the group of HR+ patients, a high sTIL score before NACT (*p* = 0.028) remained a significant predictor of longer OS, while a higher residual cancer cellularity (*p* = 0.018) and an increase of sTIL after NACT (*p* = 0.033) were associated with shorter DFS. In the HER2+ cohort, an increase of sTIL after NACT was associated with a worse DFS (*p* = 0.048), and for TN patients only a high pre-NACT sTIL was correlated with a longer OS (*p* = 0.028). 

## 4. Discussion

In this explorative study, we assessed the evolution of sTIL and the most commonly used peripheral immune markers (NLR and PLR) after NACT in IBC. Furthermore, we compared the changes observed in IBC patients to those observed in a group of subtype-matched nIBC patients. We evaluated sTIL in the pre-NACT biopsy and the post-NACT resection specimen but excluded the patients with pCR since they all had less than 1% sTIL in the tumour bed area. Interestingly, other researchers found that the numbers of sTIL in BC were comparable between residual fibrous lesions (pCR) and in residual tumour lesions (no-pCR) [8]. We demonstrated—like most studies reporting on sTIL after NACT (Table 1) [10]—that sTIL tends to decrease after NACT in more patients. This was the case in both the IBC (median δsTIL: −4.5%, *p* < 0.001) and the nIBC cohort (median δsTIL: −1.25%, *p* = 0.06), but the median decrease was significantly greater in the IBC cohort (OR: 0.25, 95% CI: 0.073–0.76, *p* = 0.018). Furthermore, this decrease was significant in all IBC subtypes. 

A high number of sTIL after NACT was associated with residual cancer cellularity in our IBC cohort, possibly indicating that more remaining tumour cells could attract more infiltrating immune cells. Other researchers demonstrated the opposite, i.e., a higher number of sTIL after NACT correlated with less tumour burden indicated by tumour size or nodal status [8,9]. This might possibly explain why in many studies a higher number of sTIL was also associated with a better prognosis [8,9,24], contrary to our findings. Indeed, in our IBC cohort a high number of sTIL after NACT correlated with a shorter OS (HR: 0.23, 95% CI: 0.05–1.02, *p* = 0.05) and shorter DFS (HR: 0.33, 95% CI: 0.11–0.98, *p* = 0.046). Interestingly, while we showed an association between cancer cellularity and number of sTIL after NACT, both were independent prognostic markers in the multivariate model. Thus, it seems that the prognostic effect of the sTIL in our IBC cohort is not only a mere reflection of tumour burden. Of course, the residual tumour cellularity is not the only marker of the residual tumour burden or response to NACT. Asano et al. also suggested that a combination of RCB and sTIL is a more sensitive predictor for DFS than sTIL alone [19]. Unfortunately, we could not calculate an RCB score for the historic cohort of IBC patients because some historical data were lacking in the pathology reports. The only other study looking at the evolution of sTIL after NACT in IBC was shown in a cohort of 31 TN IBC patients that the median post-NACT sTIL score was lower than before NACT (1.5% vs. 10%). They also demonstrated that an increase in sTIL after NACT was associated with a worse DFS (21 months vs. 101 months; *p* = 0.0002) [11]. Why some studies showed a prognostic beneficial effect can depend on a number of things. First, there is a lot of heterogeneity across studies. Different cut-off values to define higher sTIL numbers are used, some studies look at the change or δsTIL while others look solely at post-NACT sTIL, and not all patients received the same chemotherapy regimen (Table 1). Furthermore, the molecular subtype of samples, the timing of surgery and the composition of the sTIL infiltrate can also explain some differences. 

The beneficial effect of post-NACT sTIL was mostly demonstrated TNBC patients. Hamy et al. also found an association between post-NACT sTIL and a more residual tumour burden in HER2+ BC and reported worse outcomes in patients with more sTIL [10]. Other researchers saw no effect of sTIL after NACT on DFS in HER2+ BC but a borderline significant effect in TNBC [23]. Furthermore, it seems that the HR status also has an effect on the number of sTIL [12], but we could only demonstrate an association between HR status and a larger δsTIL in univariate analysis. In our study, an increase of sTIL was associated with a shorter DFS in both the HR+ and HER2+ IBC cohorts, but not in the TN IBC cohort, even though the patient numbers to do the analysis for the different molecular subtypes were small. Moreover, the composition of the immune infiltrate and the functional state of the immune cells influences the immune response against the tumour. A high number of CD8+ cells was shown to be beneficial both in terms of response to chemotherapy and survival [30], while a more regulatory immune response with FOXP3+ cells was associated with a worse outcome [31]. Furthermore, chemotherapy will induce changes in the immune profile and function of the immune cells [32]. Rufell et al. showed, for example, an increase in CD8+ cells and a decrease in CD20+ lymphocytes after chemotherapy [33]. In the study of Gracia-Martinez et al., the patients with high sTIL after NACT had a worse DFS, which was partially explained by the presence of many CD68+ macrophages that have been associated with tumour progression [32,34]. The negative prognostic effect of sTIL after NACT and the stronger decrease in sTIL in IBC might therefore be explained by a different composition of the immune infiltrate compared to nIBC disease. Further research to examine the composition and role of the different immune cells in IBC is important [35] (In press). Finally, the antitumour immune response also changes in time. The composition of the immune infiltrate in in situ, early and late-stage disease is different [36]. Therefore, the moment of surgery and the period since the last chemotherapy session might also influence the number and composition of the immune infiltrate and therefore also the prognostic effect. 

Besides the effect of NACT on the local immune infiltrate, we also examined the evolution of peripheral circulating immune cells in IBC. An elevated NLR [13], an elevated PLR [15,16] and a lower LMR [17], are all shown to be peripheral inflammation markers that are associated with a worse outcome in breast cancer. Moreover, in IBC, an elevated NLR or PLR before NACT predicts a worse prognosis. Furthermore, it seems that the elicited peripheral immune response in IBC is similar to the response in nIBC [20]. After NACT, both the NLR and the PLR decreased, while the LMR increased, but the size of these changes was not significantly associated with IBC disease. Previous studies demonstrated the (transient) myelosuppressive and immunomodulatory effects of anthracycline-taxane-based chemotherapy on the peripheral leukocytes [37,38]. While the decrease in NLR and PLR or the increase of LMR might indicate a more favorable peripheral immune profile against the tumour, we could not demonstrate an association between prognosis and the peripheral immune markers after chemotherapy. The reasons for this can be manifold. We have no idea of the composition or the functional state of the peripheral leukocytes; the effect of chemotherapy will be different according to molecular subtype and stage, the transient effects of the therapy that change over time or a more substantial reserve of progenitor stem cells in the bone marrow in some patients. Further research into a larger patient cohort will be needed to examine the prognostic role of these markers after NACT.

In this study, we managed to explore the evolution of sTIL and peripheral immune markers in a relatively large cohort of a rare form of breast cancer and compare this with a molecular subtype-matched cohort of nIBC patients. We showed that a low number of sTIL after NACT was associated with a longer DFS and that sTIL tended to decrease stronger in IBC compared to nIBC. 

However, the sample size was not large enough to do robust molecular subtype-specific analysis. Other limitations include the retrospective character, the double-center design and the fact that there are some missing or incomplete data. Therefore, it would be interesting to confirm these findings in a larger study within the context of the IBC International Consortium. 

## 5. Conclusions

There was no significant difference in the pre- and posttreatment sTIL score between IBC and nIBC, and in both groups the number of sTIL was significantly lower after NACT. However, the IBC phenotype did correlate with a stronger decrease of sTIL after NACT (OR: 0.25, 95% CI: 0.073–0.76, *p* = 0.018). In the group of IBC patients without pCR after NACT, a high number of sTIL before NACT (HR: 0.23, 95% CI: 0.05–1.02, *p* = 0.05) was prognostic for a longer OS, while a low number of sTIL after NACT (HR: 0.33, 95% CI: 0.11–0.98, *p* = 0.046) was associated with a longer DFS, besides a low residual cancer cellularity (HR: 0.20, 95% CI: 0.08–0.52, *p* < 0.001). 

## Figures and Tables

**Table 2:** Categorical clinicopathological parameters. Comparison between nIBC and IBC was done using a Chi-square test. nIBC: non-inflammatory breast cancer, IBC: inflammatory breast cancer, RD: residual disease, pCR: complete pathological response, sTIL: stromal tumour infiltrating lymphocytes and NACT: neo-adjuvant chemotherapy. * median value used for dichotomization. Bold values denote statistical significance at the *p* < 0.05 level.

Parameters	nIBC	IBC	*p*-Value
**Hormonal receptor state**			0.57
Negative	48	50	
Positive	86	75	
**HER2 state**			0.91
Negative	86	74	
Positive	48	44	
**Receptor subtypes**			0.16
HR+/HER2−	50	48	
HR+/HER2+	36	24	
HR−/HER2+	12	20	
HR−/HER2−	36	26	
**Differentiation grade**			**0.001**
Well	12	6	
Moderate	47	35	
Poor	42	79	
**Stage**			**<0.001**
Stage I or II	103	0	
Stage III	28	125	
**Response to NACT**			0.87
RD	78	75	
pCR	56	50	
Pre-NACT sTIL (cat) (%) *			0.24
<12.5%	62	68	
≥12.5%	72	57	
Post-NACT sTIL (cat) in RD (%) *			0.54
<5%	42	24	
≥5%	37	28	
**Difference in sTIL before and after NACT**			**0.034**
Increase (>5%)	13	7	
No change (≥−5–≤5%)	48	22	
Decrease (<−5%)	18	23	
**Change in sTIL after NACT**			**0.044**
Increase/stable (≥0%)	33	12	
Decrease (<0%)	46	40	

**Table 3:** Continuous clinicopathological parameters. Comparison between nIBC and IBC was done using Wilcoxon signed rank test. nIBC: non-inflammatory breast cancer, IBC: inflammatory breast cancer, RD: residual disease, NLR: neutrophil-lymphocyte ratio, PLR: platelet-lymphocyte ratio, LMR: lymphocyte-monocyte ratio and, sTIL: stromal tumour infiltrating lymphocytes, NACT: neo-adjuvant chemotherapy. Bold values denote statistical significance at the *p* < 0.05 level.

Parameters	nIBC	n	IBC	n	*p*-Value
Age (years)	53.3 (27.2–82.4)	134	56.6 (33.3–83)	125	0.087
Residual Cancer Cellularity	20 (1–90)	70	15 (1–90)	52	0.38
NLR (Moment of diagnosis)	2.68 (0.86–12.9)	129	2.53 (0.85–9.11)	56	0.46
PLR (Moment of diagnosis)	150 (61–569)	129	150 (73–299)	56	0.79
LMR (Moment of diagnosis)	4.35 (0.69–23.7)	129	3.43 (1–9.5)	56	**<0.001**
NLR (After NACT)	4.02 (0.28–60)	86	3.43 (0.04–21.0)	42	0.11
PLR (After NACT)	274 (43–1006)	86	204 (51.5–840)	42	**0.005**
LMR (After NACT)	1.74 (0.52–13.9)	86	2.28 (0.79–7.2)	39	**0.03**
Pre-NACT sTIL (%)	10 (1–85)	134	12.5 (1–80)	125	0.13
Post-NACT sTIL in RD (%)	5 (1–60)	79	4 (1–90)	52	0.16
Difference in sTIL before and after NACT (%)	−1.25 (−81–32.5)	79	−4.5 (−48–75)	52	**0.018**

**Table 4:** Uni- and multivariate analysis for decrease of sTIL after NACT (<−2.5%). nIBC: non-inflammatory breast cancer, IBC: inflammatory breast cancer, HR: hormone receptor status, sTIL: stromal tumour infiltrating lymphocytes, NACT: neo-adjuvant chemotherapy. RD: residual disease, NLR: neutrophil-lymphocyte ratio, PLR: platelet-lymphocyte ratio, LMR: lymphocyte-monocyte ratio and, sTIL: stromal tumour infiltrating lymphocytes, NACT: neo-adjuvant chemotherapy. Bold values denote statistical significance at the *p* < 0.05 level.

Parameters	Univariate Analysis		Multivariate Analysis	
	OR (95% CI)	*p*-Value	OR (95% CI)	*p*-Value
nIBC vs. IBC	0.23 (0.106–0.481)	**<0.001**	0.247 (0.073–0.761)	**0.02**
HR− vs. HR+	2.174 (1.019–4.756)	**0.047**	2.277 (0.729–7.43)	0.16
HER2− vs. HER2+	0.749 (0.333–1.667)	0.479		
Differentiation: Low vs. Moderate Low vs. High	0.847 (0.246–2.801) 0.643 (0.188–2.11)	0.787 0.47		
sTIL pre-NACT: <12.5% vs. ≥12.5%	0.099 (0.04–0.223)	**<0.001**	0.022 (0.003–0.095)	**<0.001**
sTIL post-NACT: <5% vs. ≥5%	2.032 (1.018–4.115)	**0.046**	13.12 (3.181–93.55)	**0.002**
Nodal status: cN0/1 vs. cN2/3	0.886 (0.389–2.028)	0.774		
Cellularity: <20% vs. >20%	2.021 (0.963–4.31)	0.065		
NLR: <2.64 vs. ≥2.64	1.832 (0.854–3.982)	0.122		
PLR: <150 vs. ≥ 150	2.289 (1.062–5.027)	**0.036**	2.005 (0.730–5.673)	0.18
LMR: <4.05 vs. ≥ 4.05	1.909 (0.898–4.107)	0.094		
NLR after NACT: <2.64 vs. ≥2.64	1.059 (0.499–2.252)	0.879		
PLR after NACT: <250 vs. ≥250	1.595 (0.649–4.127)	0.318		
LMR after NACT: <1.82 vs. ≥1.82	1.158 (0.445–3.017)	0.762		
Age: <54.45 vs. ≥54.45	0.611 (0.226–1.616)	0.324		

**Table 5:** Uni- and multivariate analysis: Clinicopathological parameters associated with a higher number of sTIL after NACT in the IBC cohort. IBC: inflammatory breast cancer, HR: hormone receptor status, sTIL: stromal tumour infiltrating lymphocytes, NACT: neo-adjuvant chemotherapy. RD: residual disease, NLR: neutrophil-lymphocyte ratio, PLR: platelet-lymphocyte ratio and LMR: lymphocyte-monocyte ratio. Bold values denote statistical significance at the *p* < 0.05 level.

Parameters	Univariate Analysis		Multivariate Analysis	
	OR (95% CI)	*p*-Value	OR (95% CI)	*p*-Value
HR− vs. HR+	0.472 (0.145–1.473)	0.201		
HER2− vs. HER2+	0.694 (0.181–2.473)	0.578		
Differentiation: Low vs. Moderate Low vs. High	1.199 (0.093–29.14) 2.428 (0.211–55.54)	0.891 0.487		
sTIL pre-NACT: <12.5% vs. ≥12.5%	2.999 (0.987–9.648)	**0.057**	2.13 (0.51–9.73)	0.30
Nodal status: cN0/1 vs. cN2/3	2.142 (0.583–8.317)	0.257		
Cellularity: <20% vs. >20%	11.519 (3.03–52.582)	**0.001**	11.64 (2.99–55.29)	**<0.001**
NLR: <2.64 vs. ≥2.64	1.333 (0.327–5.607)	0.688		
PLR: <150 vs. ≥ 150	1.285 (0.318–5.298)	0.723		
LMR: <4.05 VS. ≥4.05	1.4 (0.34–6.058)	0.642		
Age: <54.45 vs. ≥54.45	1.111 (0.29–4.272)	0.877		
NLR after NACT: <2.64 vs. ≥2.64	1.23 (0.225–7.353)	0.810		
PLR after NACT: <250 vs. ≥250	1.296 (0.23–8.148)	0.770		
LMR after NACT: <1.82 vs. ≥1.82	2.999 (0.437–27.129)	0.279		
PDL1: <1% vs. >1%	1.217 (0.606–2.493)	0.58		

**Table 6:** Uni- and multivariate analysis for OS in the group of IBC patients without pCR after NACT. IBC: inflammatory breast cancer, HR: hormone receptor status, sTIL: stromal tumour infiltrating lymphocytes, NACT: neo-adjuvant chemotherapy, NLR: neutrophil-lymphocyte ratio, PLR: platelet-lymphocyte ratio and LMR: lymphocyte-monocyte ratio. Bold values denote statistical significance at the *p* < 0.05 level.

Parameters	Univariate Analysis		Multivariate Analysis	
	HR (95% CI)	*p*-Value	HR (95% CI)	*p*-Value
Age: <56.6 vs. ≥56.6	1.86 (0.75–4.61)	0.17		
HR− vs. HR+	0.54 (0.25–1.13)	0.11		
HER2− vs. HER2+	1.49 (0.65–3.41)	0.34		
Differentiation: Moderate vs. High	0.88 (0.4–1.89)	0.74		
Nodal status: cN0/1 vs. cN2/3	2.97 (1.13–7.81)	**0.03**	1.93 (0.64–5.80)	0.24
PDL1: <1% vs. >1%	0.91 (0.56–1.50)	0.72		
TIL pre-NACT: <10% vs. ≥10%	2.08 (0.98–4.41)	**0.05**	4.47 (1.37–14.5)	**0.01**
TIL post-NACT: <4% vs. ≥4%	0.48 (0.22–1.04)	**0.06**	0.23 (0.05–1.02)	**0.05**
Cellularity: <17.5% vs. ≥17.5%	0.23 (0.09–0.53)	**<0.001**	0.67 (0.20–2.19)	0.50
NLR: <2.4 vs. ≥2.4	1.42 (0.57–3.53)	0.44		
PLR: <163 vs. ≥163	1.09 (0.44–2.71)	0.84		
LMR: <3.6 vs. ≥3.6	0.93 (0.36–2.38)	0.88		
NLR after NACT: <3.3 vs. ≥3.3	1.31 (0.43–4.01)	0.63		
PLR after NACT: <171 vs. ≥171	1.27 (0.33–4.84)	0.71		
LMR after NACT: <2.6 vs. ≥2.6	0.27 (0.07–1.12)	0.07		
Change: increase vs. decrease	1.85 (0.80–4.28)	0.15		

**Table 7:** Uni- and multivariate analysis for DFS in the group of IBC patients without pCR after NACT. IBC: inflammatory breast cancer, HR: hormone receptor status, sTIL: stromal tumour infiltrating lymphocytes, NACT: neo-adjuvant chemotherapy, NLR: neutrophil-lymphocyte ratio, PLR: platelet-lymphocyte ratio and LMR: lymphocyte-monocyte ratio.

Parameters	Univariate Analysis		Multivariate Analysis	
	HR (95% CI)	*p*-Value	HR (95% CI)	*p*-Value
Age: <56.6 vs. ≥56.6	1.88 (0.82–4.32)	0.13		
HR− vs. HR+	0.53 (0.26–1.08)	0.08		
HER2− vs. HER2+	1.17 (0.52–2.64)	0.7		
Differentiation: Moderate vs. High	1.17 (0.57–2.38)	0.66		
Nodal status: cN0/1 vs. cN2/3	1.83 (0.80–4.22)	0.15		
PDL1: <1% vs. > 1%	1.13 (0.75–1.68)	0.56		
TIL pre-NACT: <10% vs. ≥10%	1.28 (0.63–2.61)	0.49		
TIL post-NACT: <4% vs. ≥4%	0.31 (0.14–0.68)	**0.003**	0.33 (0.11–0.98)	**0.046**
Cellularity: <17.5% vs. ≥17.5%	0.14 (0.06–0.33)	**<0.001**	0.20 (0.08–0.52)	**<0.001**
NLR: <2.4 vs. ≥2.4	1.38 (0.61–3.14)	0.43		
PLR: <163 vs. ≥163	1.47 (0.65–3.33)	0.35		
LMR: <3.6 vs. ≥3.6	0.48 (0.21–1.11)	0.09		
NLR after NACT: <3.3 vs. ≥3.3	1.38 (0.54–3.50)	0.49		
PLR after NACT: <171 vs. ≥171	2.32 (0.75–7.15)	0.13		
LMR after NACT: <2.6 vs. ≥2.6	0.54 (0.15–1.92)	0.34		
Change: increase vs. decrease	2.26 (1.03–4.96)	0.04	2.08 (0.85–5.13)	0.11

## Data Availability

The datasets generated during and/or analyzed during the current study are not publicly available due individual patient privacy but are available from the corresponding author on reasonable request.

## Supplementary Materials

The following are available online at https://www.mdpi.com/article/10.3390/cancers13184656/s1, Figure S1: Evolution of sTIL after NACT in individual nIBC patients. Figure S2: Median sTIL score before NACT in the patients with pCR compared to patients with RD. Figure S3: Kaplan–Meier curves for DFS and the change in sTIL in IBC. Table S1: Clinicopathological parameters associated with a higher number of sTIL after NACT (>5%) in the total patient cohort. Table S2: Clinicopathological parameters associated with a higher number of sTIL after NACT in the nIBC cohort. Table S3: Clinicopathological parameters associated with pCR after NACT in IBC.
