# Supplementary material for: The Evolution and Prognostic Role of Tumour-Infiltrating Lymphocytes and Peripheral Blood-Based Biomarkers in Inflammatory Breast Cancer Patients Treated with Neoadjuvant Chemotherapy
Source: Cancers (Basel). 2021 Sep 16;13(18):4656. doi: 10.3390/cancers13184656 (PMC8471511; doi:10.3390/cancers13184656)
Supplement: Supplementary file 1 [file cancers-13-04656-s001.zip › cancers-1263032-supplementary.pdf]

SUPPLEMENTARY FIGURES AND TABLES

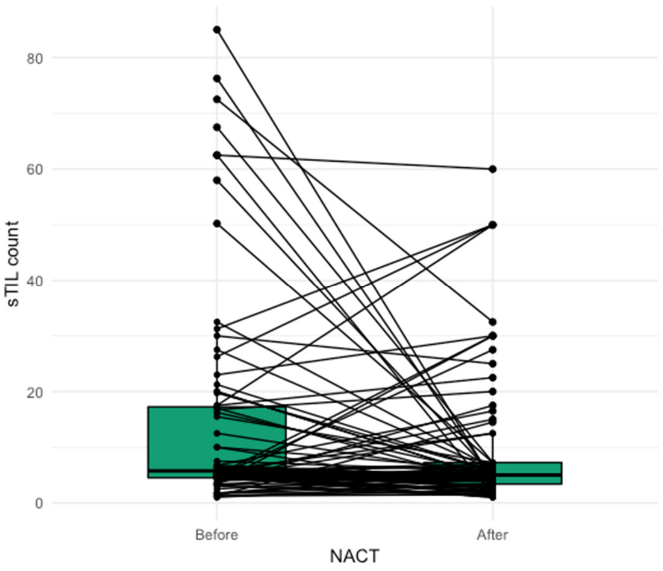

Figure S1: Evolution of sTIL after NACT in individual nIBC patients. Out of 79 patients, 13 had an increase, 48 had no change and 18 patients had a decrease. nIBC: non-inflammatory breast cancer, sTIL: stromal tumour infiltrating lymphocytes, NACT: neo-adjuvant chemotherapy.

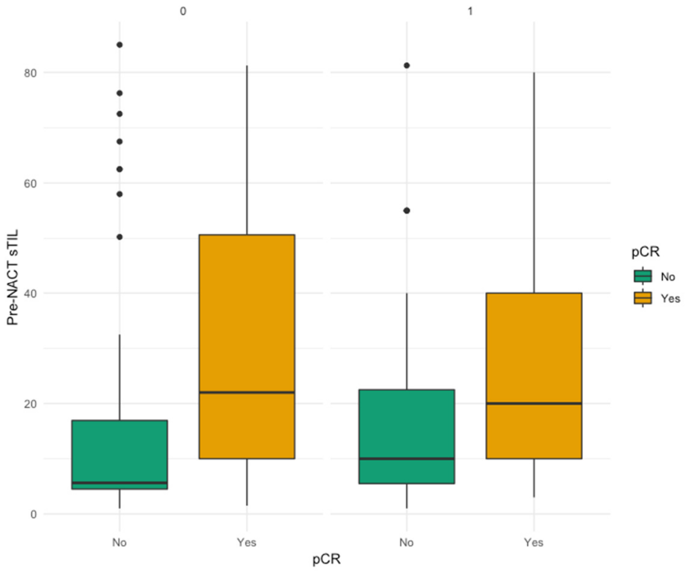

Figure S2: There is a significantly higher median sTIL score before NACT in the patients with pCR compared to patients with RD in both nIBC (left) and IBC (right) patients. nIBC: 5.5 (1 – 85) vs. 22 (1.5 – 81), P< 0.001, IBC: 10 (1 – 55) vs. 20 (3 – 80), P< 0.001. sTIL: stromal tumour infiltrating lymphocytes, RD: residual disease, pCR: complete pathological response.

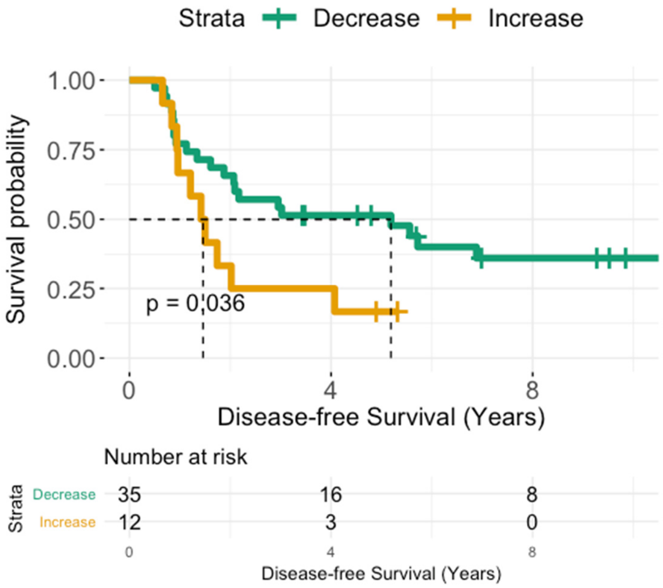

Figure S3: Kaplan-Meier curves for DFS and the change in sTIL in IBC. Patients with a decrease have a significant longer DFS: Median survival of 5.19 year (95%CI 2.07 – NR) vs. 1.46 year (95%CI 0.96 – NR).

Table S1: Uni- and multivariate analysis: Clinicopathological parameters associated with a higher number of sTIL after NACT(> 5%) in the total patient cohort. nIBC: non-inflammatory breast cancer, IBC: inflammatory breast cancer, HR: hormone receptor status, sTIL: stromal tumour infiltrating lymphocytes, NACT: neo-adjuvant chemotherapy. RD: residual disease, NLR: neutrophil-lymphocyte ratio, PLR: platelet-lymphocyte ratio and LMR: lymphocyte-monocyte ratio

Parameters	Univariate Analysis		Multivariate Analysis	
	OR (95% CI)	P-value	OR (95% CI)	P-value
nIBC vs. IBC	0.755 (0.372 - 1.521)	0.433		
HR- vs. HR+	0.525 (0.24 - 1.118)	0.099		
HER2- vs. HER2+	0.716 (0.316 - 1.595)	0.416		
Differentiation: Low vs. Moderate<br>Low vs. High	1.139 (0.339 - 4.186)<br>3.3 (0.99 – 12.1)	0.835<br>0.06		
sTIL pre-NACT: <12.5% vs. ≥12.5%	3.253 (1.569 - 6.97)	**0.002**	2.66 (1.19 – 6.20)	**0.019**
Nodal status: cN0/1 vs. cN2/3	1.461 (0.642 - 3.399)	0.369		
Cellularity: < 20% vs. >20%	3.412 (1.6 - 7.495)	**0.002**	3.50 (1.60 – 7.89)	**0.002**
NLR: <2.64 vs. ≥2.64	1.372 (0.645 - 2.94)	0.411		
PLR: <150 vs. ≥ 150	1.094 (0.513 - 2.334)	0.816		
LMR: < 4.05 VS. ≥ 4.05	1.509 (0.714 - 3.222)	0.282		
Age: <54.45 vs. ≥ 54.45	0.962 (0.454 - 2.033)	0.920		
NLR after NACT: < 2.64 vs. ≥2.64	1.034 (0.426 - 2.532)	0.941		
PLR after NACT: < 250 vs. ≥ 250	0.804 (0.305 - 2.093)	0.657		
LMR after NACT: <1.82 vs. ≥ 1.82	1.277 (0.483 - 3.406)	0.621		

Table S2: Uni- and multivariate analysis: Clinicopathological parameters associated with a higher number of sTIL after NACT in the nIBC cohort. IBC: inflammatory breast cancer, HR: hormone receptor status, sTIL: stromal tumour infiltrating lymphocytes, NACT: neo-adjuvant chemotherapy. RD: residual disease, NLR: neutrophil-lymphocyte ratio, PLR: platelet-lymphocyte ratio and LMR: lymphocyte-monocyte ratio.

Parameters	Univariate Analysis		Multivariate Analysis	
	HR (95% CI)	P-value	OR (95% CI)	P-value
HR- vs. HR+	0.853 (0.662 - 1.1)	0.225		
HER2- vs. HER2+	0.926 (0.714 - 1.201)	0.567		
Differentiation: Low vs. Moderate<br>Low vs. High	1.137 (0.282 – 5.066)<br>7.438 (1.53 – 43.30)	0.858<br>**0.016**	1.10 (0.26 – 5.19)<br>5.42(1.03 – 33.6)	0.9035<br>0.053
sTIL pre-NACT: <12.5% vs. ≥12.5%	1.396 (1.112 - 1.753)	**0.005**	4.33 (1.22 – 18.0)	**0.03**
Nodal status: cN0/1 vs. cN2/3	1.062 (0.778 - 1.449)	0.705		
Cellularity: < 20% vs. >20%	1.146 (0.905 - 1.45)	0.260		
NLR: <2.64 vs. ≥2.64	1.086 (0.865 - 1.364)	0.475		
PLR: <150 vs. ≥ 150	1.005 (0.8 - 1.262)	0.963		
LMR: < 4.05 VS. ≥ 4.05	1.119 (0.894 - 1.402)	0.327		
Age: <54.45 vs. ≥ 54.45	0.972 (0.774 - 1.222)	0.814		
NLR after NACT: < 2.64 vs. ≥2.64	0.991 (0.763 - 1.288)	0.951		
PLR after NACT: < 250 vs. ≥ 250	0.897 (0.659 - 1.222)	0.497		
LMR after NACT: <1.82 vs. ≥ 1.82	1 (0.738 - 1.354)	1.000		

Table S3: Uni- and multivariate analysis: Clinicopathological parameters associated with pCR after NACT in IBC. IBC: inflammatory breast cancer, HR: hormone receptor status, sTIL: stromal tumour infiltrating lymphocytes, NACT: neo-adjuvant chemotherapy, NLR: neutrophil-lymphocyte ratio, PLR: platelet-lymphocyte ratio and LMR: lymphocyte-monocyte ratio.

Parameters	Univariate Analysis		Multivariate Analysis	
	OR (95% CI)	P-value	OR (95% CI)	P-value
HR- vs. HR+	0.829 (0.695 – 0.989)	**0.04**	0.47 (0.21 – 1.07)	0.07
HER2- vs. HER2+	1.263 (1.051 - 1.519)	**0.014**	2.61 (1.15 – 6.09)	**0.02**
Differentiation: Moderate vs. High	0.92 (0.763 - 1.11)	0.388		
sTIL pre-NACT: <12.5% vs. ≥12.5%	1.283 (1.081 - 1.523)	**0.005**	2.32 (0.97 – 5.77)	**0.06**
Nodal status: cN0 vs. cN+	0.942 (0.838 - 1.06)	0.332		
Cellularity: < 20% vs. >20%	0.983 (0.758 - 1.274)	0.900		
PDL1: < 1% vs. > 1%	1.167 (1.056 – 1.290)	**0.003**	1.46 (0.88 – 2.48)	0.14

NLR: <2.64 vs. ≥2.64	1.052 (0.807 - 1.372)	0.705		
PLR: <150 vs. ≥ 150	0.91 (0.698 - 1.185)	0.489		
LMR: < 4.05 vs. ≥ 4.05	1.01 (0.77 – 1.32)	0.96		
Age: <54.45 vs. ≥ 54.45	0.971 (0.751 - 1.255)	0.825		
